# Spatial analysis of femtosecond laser generated plasma using principal component analysis

**DOI:** 10.1038/s41598-024-81389-9

**Published:** 2024-12-05

**Authors:** James A. Grant-Jacob, Michalis N. Zervas, Ben Mills

**Affiliations:** Optoelectronics Research Centre, University of Southampton, Southampton, UK

**Keywords:** Laser material processing, Ultrafast lasers, Imaging and sensing

## Abstract

The appearance of plasma generated during femtosecond laser machining depends strongly on the features present on the sample before machining occurs. However, the complexity of femtosecond light-matter interaction means that development of a theoretical understanding of plasma generation is challenging. In this work, principal component analysis is applied to experimental images of plasma generated during femtosecond laser machining of silicon to calculate the orthogonal spatial patterns of the plasma variance (plasma modes), and to identify which sample variance (sample modes) are associated with these plasma modes. The results demonstrate the potential of principal component analysis for data-driven scientific discovery in the field of femtosecond light-matter interactions.

## Introduction

Femtosecond laser machining has emerged as a powerful tool in modern manufacturing, offering unprecedented precision and versatility in material processing^1–3^. During femtosecond laser machining, intense light pulses interact with the target material, leading to the formation of plasma through multiphoton ionisation and subsequent ablation processes^4^. Understanding the dynamics of the generated plasma is crucial for optimising machining parameters and ensuring the quality of fabricated structures, and for helping to understand the nonlinear nature of light-matter interaction on the femtosecond timescales. Whilst there have been studies of femtosecond-generated plasma that start from a theoretical understanding^5,6^, recent results in the field of physics have highlighted the importance of data-driven analysis, where instead a process is understood and optimised directly from the experimental data and often without the need to make any theoretical assumptions^7^. Deep learning^8^ is a data-driven analytical tool that has been used extensively for data-driven analysis and optimisation of laser materials processing^9–13^. Indeed, recent results have shown the application of neural networks for identification of sample features from images of plasma^14–16^, the application of plasma for real-time control of laser machining^17^, and for processing data from a range of laser-physics experiments^18^. However, the opaque nature of neural networks can make it challenging to uncover the fundamental properties, and hence uncover novel understanding, for such processes. There is therefore an advantage in using a data-driven analytical approach that also provides a mathematical output that be used to help further develop scientific understanding.

Principal Component Analysis (PCA)^19^ is a mathematical technique used for dimensionality reduction and data analysis. It can transform high-dimensional data into a lower-dimensional space while retaining the most important information, by identifying orthogonal axes of variance that are known as principal components. These components provide a new basis for representing the data, and in the case of image data can be referred to as the “modes” of a system, and this is the nomenclature used here. Widely applied in various fields such as statistics, machine learning, and signal processing, PCA plays a crucial role in uncovering latent structures and reducing the complexity of datasets, thereby facilitating efficient mathematical analysis and modelling. In this work, PCA offers the potential as a data-driven (but mathematically explainable) approach for analysis of the spatial information contained within images of laser-generated plasma.

Here, PCA is applied to experimental images of plasma generated from single femtosecond pulses ablating a silicon target to identify the fundamental modes of the plasma. By also including the camera images of the sample, the PCA approach can identify associations between the features of the sample and the shape of the generated plasma. The results presented in this work therefore demonstrate the potential for using PCA for data-driven scientific discovery in the field of femtosecond light-matter interactions. This application of PCA for femtosecond laser machining may also help enhance process optimisation and unlock improved precision and efficiency in microfabrication and nanomanufacturing.

An important complementary approach to that presented here is Laser Induced Breakdown Spectroscopy (LIBS)^20,21^, which is a diagnostic technique that involves using a high-energy laser to vaporise and excite a sample, hence generating a plasma. Analysis of the spectrum of the plasma then allows the identification of the elemental composition of the sample, and indeed PCA has also been applied to LIBS^22–24^. However, critically, whilst LIBS uses the spectral information from the plasma to identify the material being ablated, the technique presented in this work unlocks analysis of the spatial information of the plasma to provide insight into the surface features on the sample. Indeed, there is undoubtedly synergistic benefit if both techniques were to be used simultaneously.

### Experimental setup

**Fig. 1:**
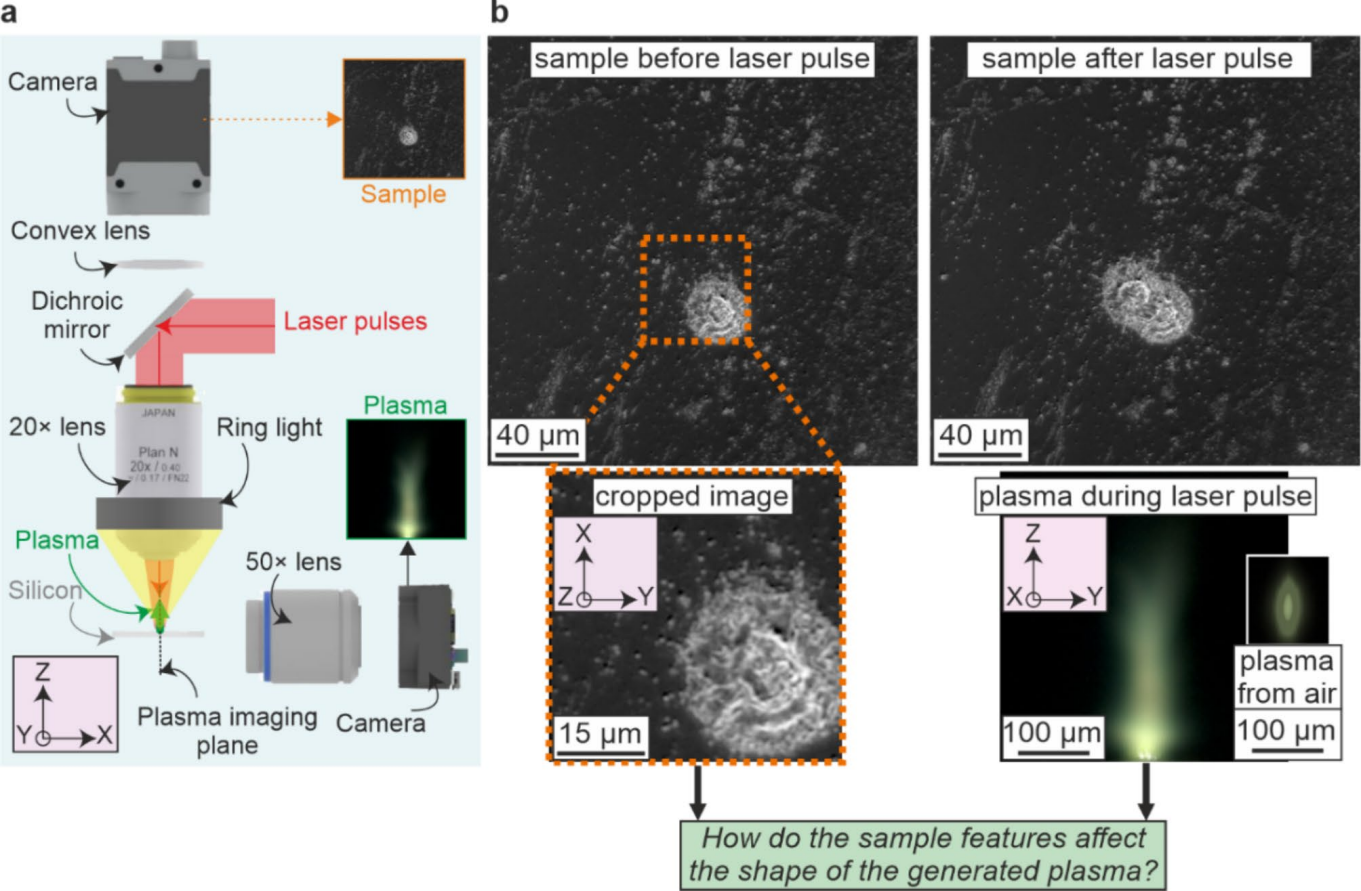
**a**) Experimental setup and **b**) example images of the sample before and after the pulse, along with the associated plasma image. The green box presents the research question.

**Fig. 2:**
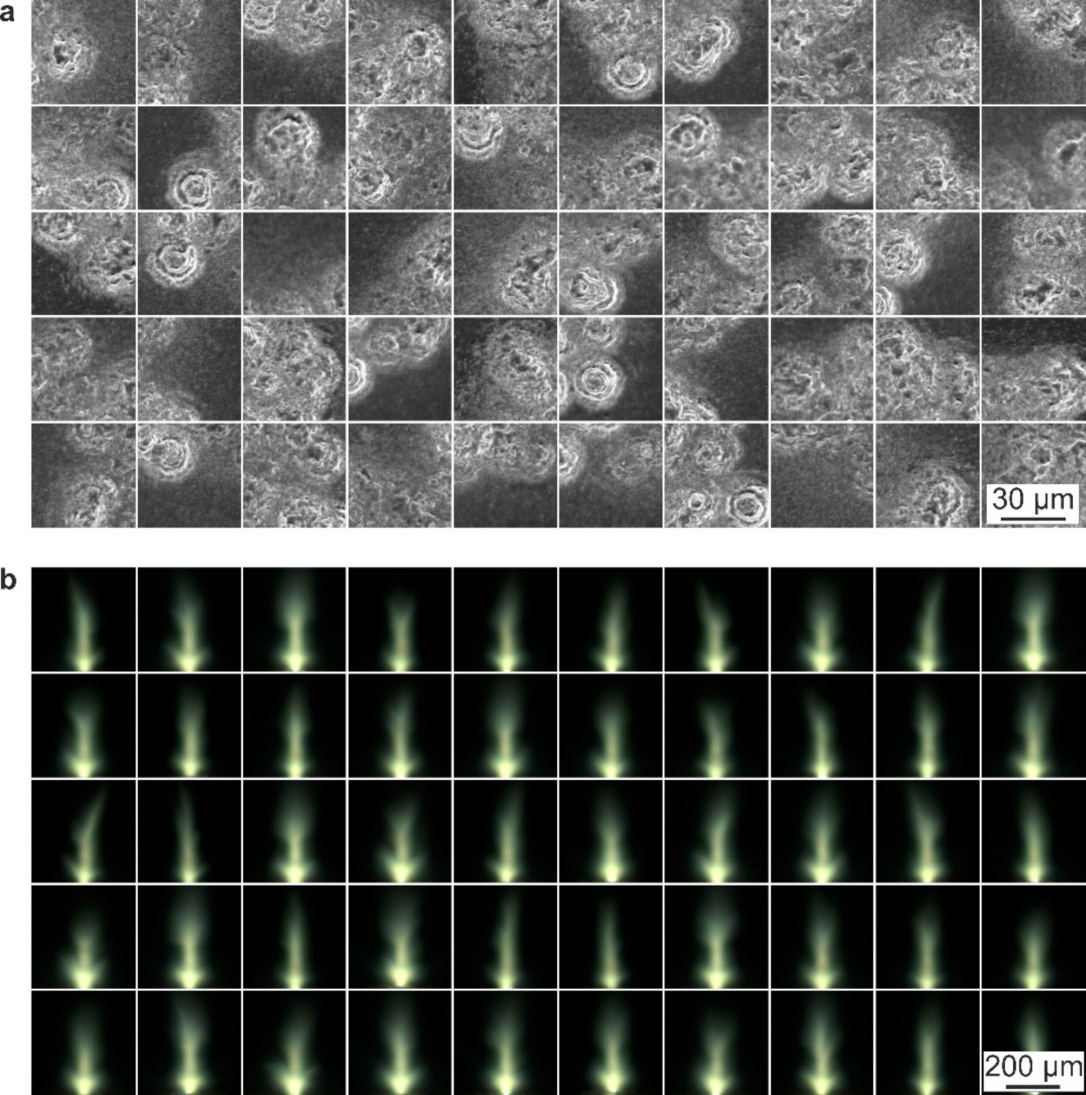
Experimental data of **a**) fifty images of the sample before the laser pulse and **b**) the fifty associated plasma images, highlighting the complex relationship between sample features and the resultant plasma.

As shown by the schematic in Fig. 1a), laser pulses from a Light Conversion Pharos SP laser (190 fs, 1 mJ, 1030 nm, and a pulse-to-pulse energy stability of < 0.5% over a 24-hour period) were focussed onto the surface of a silicon sample (p-type) using a 20× objective (Nikon, TU Plan ELWD, 0.40 NA). From observation of ablated regions on the sample, the laser pulse spatial intensity that was above the ablation threshold for the materials had a diameter of ∼30 μm. The sample was able to be translated using an XYZ motorized translation stage (Zaber, LSM050A-T4). The top surface of the silicon was imaged along the laser axis using a Basler acA4112-20uc camera (1914 × 1200, RGB) using a convex tube lens with 20 cm focal length, providing a field of view of approximately 200 × 200 μm. The emitted plasma was imaged in the perpendicular direction using a Basler daA1920-160uc camera (4096 × 3000, RGB) coupled with a 50× long working distance objective (Olympus, SLMPLN, 0.35 NA). A ring illumination device (Navitar) was mounted around the objective to allow imaging of the sample. This device featured a remote white-light source connected to the ring via an umbilical cord, with a computer-controlled shutter positioned between the remote light source and the cord. This setup ensured uniform, shadow-free illumination for darkfield imaging and allowed the illumination to be automatically blocked by the shutter when capturing plasma images. The laser, translation stages, two cameras, and the shutter were automated using Python, with the following order of action producing a single data set: translate sample to a new position, take camera image of sample, close shutter, trigger a single laser pulse, take camera image of plasma, and open shutter. Each data set therefore contained an image of the sample before the laser pulse, and an associated image of the plasma generated when a laser pulse was incident on that region of the sample. The sample was translated by a random direction and distance between each data set collection, with the randomness chosen to ensure that a high percentage of positions overlapped with previous positions. In other words, most camera images of the sample contained surface features that were created by previous laser pulses. The data collection process was repeated to give 4289 data sets, hence formed of 4289 camera images of the sample and 4289 associated plasma images. Figure 1b) show a set of example images of the sample before and after the pulse, along with the associated plasma image. To convey the variation within the experimental data, Fig. 2 shows fifty examples of images of the sample before the laser pulse, along with the fifty associated plasma images.

**Fig. 3:**
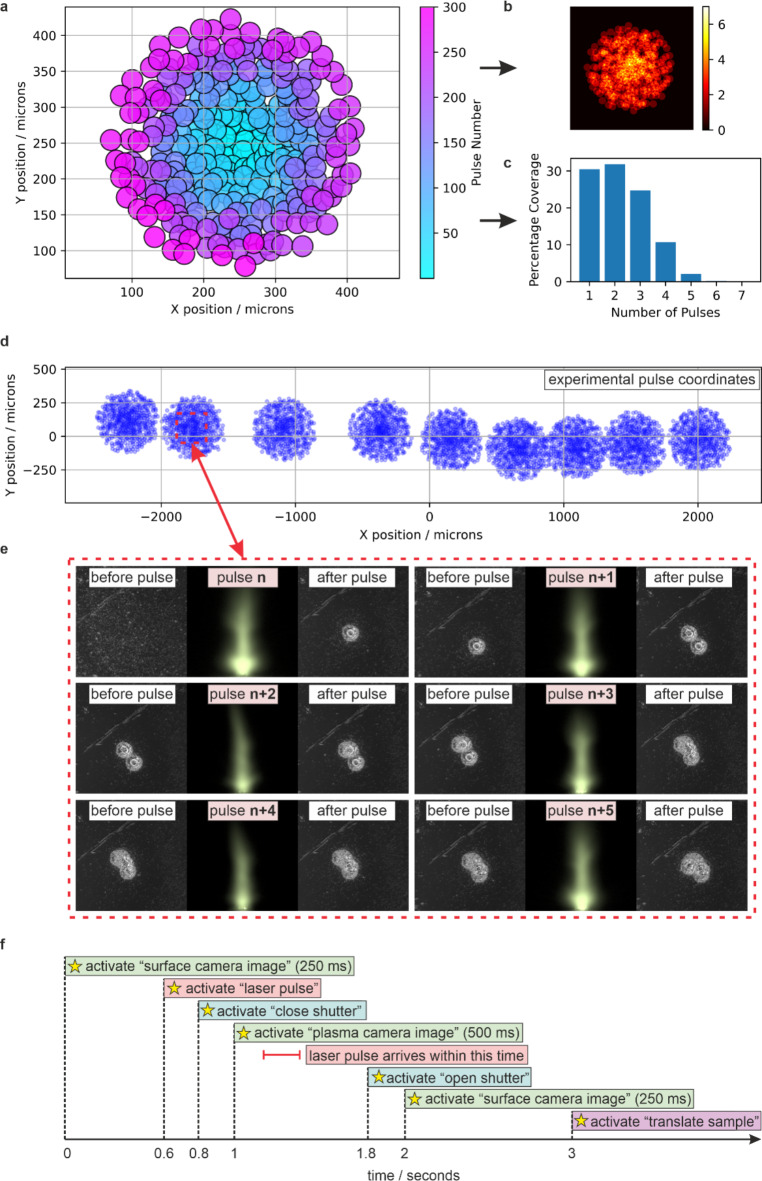
**a**) Example set of random sample coordinates for each incident pulse, **b**) associated depth map, **c**) histogram of spatial overlap, d) coordinates of all experimental laser pulses, **e**) example set of data associated with six consecutive laser pulses, and **f**) the software timings for data collection.

Figure 3 explains the method of data collection and presents (a) an example set of random sample coordinates for each incident pulse, (b) an associated depth map showing the number of pulses overlapping at each position, and (c) a histogram of this data highlighting that most of the sample is machined with 1, 2 or 3 pulses (and hence only sub-micron average depth changes occur). Figure 3 also shows (d) the coordinates of all experimental laser pulses for this work, (e) an example set of data associated with six consecutive laser pulses, and (f) the software timings for the data collection. The sample coordinates were determined by adding small deviations in X and Y, along with mean reversion, to an Archimedean spiral, as this ensured that the coordinates had a random overlap in both magnitude and direction. Each of the nine circular experimental regions contained between 450 and 500 pulses, hence resulting in 4289 laser pulses in total. The software timings were designed to take into account the uncertainty in software-hardware communication latency, and each data item collection took three seconds. Due to the uncertainty in laser pulse arrival time (a consequence of using a network-based communication protocol), an integration time of 500 ms was used for the plasma camera, which meant that temporal dynamics of the plasma were not recorded. Laser light scattered from the sample was not captured in the plasma camera image, due to the angle of imaging. Each subsequent stage translation took approximately five seconds, and therefore the total time for data collection was approximately ten hours.

### Principal component analysis

PCA is a mathematical technique employed for dimensionality reduction and data analysis. This technique can transform high-dimensional data into a lower-dimensional space while preserving the most significant information. PCA achieves this by identifying orthogonal axes of variance, which are referred to as principal components. These principal components establish a new basis for representing the data, enabling a more compact and insightful representation of the original data set. The first principal component captures the maximum variance in the data, providing a direction along which the data points are most spread out, and subsequent principal components are orthogonal to the previous ones, each capturing the next highest variance while ensuring no redundancy in the information represented. As a result, each principal component adds a new layer of insight into the structure of the data, progressively revealing patterns and correlations that might otherwise be obscured in the original high-dimensional space.

In this work, PCA is applied to image data, for both the appearance of the sample before a laser pulse, and the appearance of the plasma resulting from the laser pulse. The 4289 pairs of images of sample and plasma were combined into the three image data sets of ‘sample only’, ‘plasma only’, and ‘sample and plasma combined’. Each of these images (4289 × 3 = 12867) were converted to a single colour channel using the mean of the RGB values, cropped to 256 × 256, resized to 128 × 128, and then flattened into a 1 × 16,384 array. The principal components were calculated for all 12,867 sets of 1 × 16,384 arrays, using the sklearn.decomposition library in Python. After calculation, the 1 × 16,384 arrays were converted back into 128 × 128 arrays, and converted back to RGB images by duplicating the single channel and then applying a correction of 83%, 118% and 105% to the RGB channels to return to the original colour map. No preprocessing was applied to the images, and the PCA calculation used the following parameters: n_components = 1000, whiten = False, svd_solver=’auto’, tol = 0.0, iterated_power=’auto’, power_iteration_normalizer=’auto’, and random_state = None. In this work, each principal component is rescaled when plotting on a figure, by dividing all values by its standard deviation, so that multiple principal components can be plotted on the same colour bar scale.

### Identifying the relationship between sample and plasma

**Fig. 4:**
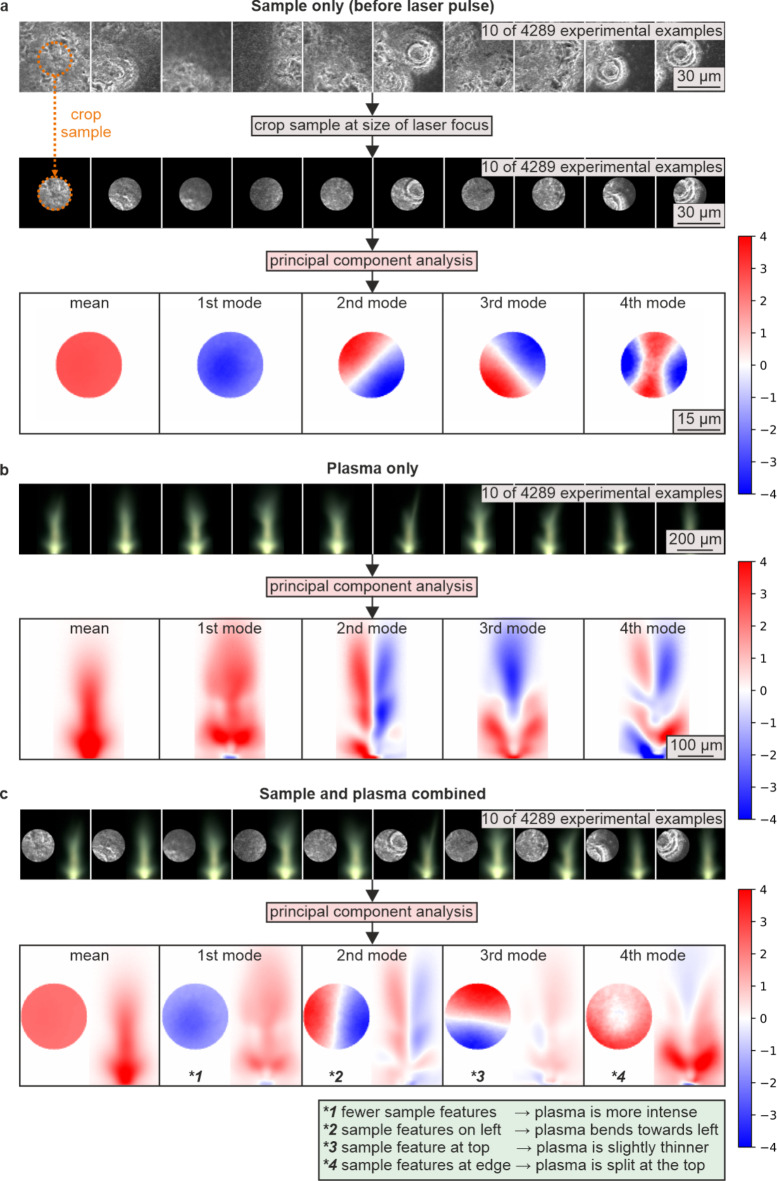
The mean and first four calculated modes for **a**) sample only, **b**) plasma only, and **c**) sample and plasma, where the modes of the combined images highlight the experimentally observed associations between the sample and plasma. The green box highlights the conclusions.

Figure 4 shows the application of PCA on experimental data, for (a) just the sample, (b) just the plasma, and (c) the combination of sample and plasma. In each case, the mean and the first four principal components are presented. Part a) also shows how the images of the sample were cropped, using a circular profile that corresponded to the spatial extent of the laser pulse, which was calculated through observation of the size and position of ablation on the sample. In each case, ten examples from the experimental dataset of 4289 examples are shown. The green box at the bottom of the figure highlights the primary conclusions.

In the images of modes, regions of red correspond to an increase in brightness of the sample and the plasma, and regions of blue to a decrease. An increase in brightness of the sample will correspond to higher white-light scattering when observed by the camera, and hence the sample brightness is associated with the amount of laser machining that has occurred at that position, prior to the arrival of the new laser pulse. An unmachined region of silicon will appear black (i.e., low brightness), and it will generally have an increasing degree of features (and hence appear brighter under white light illumination) as additional laser pulses are incident at that position. An increase in brightness of the plasma is simply a higher number of photons emitted from the plasma at each position in the image. The colour scale is arbitrary as all mode data is normalised to a standard deviation of one, and is clipped at ± 4 for readability.

Whilst the modes in (a) and (b) provide useful analysis of the distribution in appearance of the sample and plasma, the key result is associated with the combined images in c), and the following text discusses these “combined” modes in more detail. As the combined images contain both the sample and the resultant plasma, the combined modes correspond to the changes in the shape of the plasma for any given change in the sample.

The mean mode corresponds to the average sample, capturing a baseline of common features rather than representing a completely flat or featureless sample. The associated plasma response in this mean mode is highly symmetrical in the left-right plane, reflecting an average plasma behaviour across samples. The *n* = 1 mode reflects a relative decrease in the brightness of the sample, with slight emphasis around the centre. This mode can be interpreted as a contribution from samples that have fewer or subtler machined features compared to the mean. The resulting plasma image shows an increased intensity with a largely symmetrical left-right plasma response. The *n* = 2 mode captures an asymmetry in the brightness of the sample, where one side (left or right) shows higher brightness relative to the other. This indicates a sample that has been more strongly machined on one side, leading to a corresponding increase in plasma brightness on the more machined side. For example, a higher brightness on the left corresponds to greater plasma brightness on the left, and the opposite is true for higher brightness on the right. The *n* = 3 mode represents a difference in brightness between the top and bottom of the sample, and suggests that when the top of the sample is brighter, the plasma appears slightly taller with a narrower neck. These conventions apply when the corresponding component value is positive, and their interrelations reverse when the corresponding component value is negative.

The fact that the *n* = 3 changes are more subtle than the case for *n* = 2 is understandable, due to the orientation of imaging by the camera, where the normal to the camera surface is perpendicular to the left-right plane but parallel to the top-bottom plane (see Fig. 1 for a description of orientations). The *n* = 4 mode shows that a predominantly circularly symmetric sample that is more strongly machined in the outer region will result in a left/right symmetric plasma that has considerably brighter bottom wings and dimmer top, hence resulting in a plasma that is split at the top. Higher order modes show increasingly granular changes to the sample and the resultant effect on the shape of the plasma, and therefore “higher order” associations can be determined. The results indicate that the combined PCA modes are not a straightforward combination of the same order of sample-only and plasma-only PCA modes. While the mean, 1st, and 2nd combined PCA modes are combinations of the corresponding PCA modes of sample-only and plasma-only images, the 3rd and 4th combined PCA modes comprise of newly appearing plasma modes. Given that the PCA mode order reflects the relative significance of the mode in the variation of the original data, the observed re-arranging of the combined PCA modes is further indication that the combined PCA mode analysis likely unveils hidden relations between sample features and plasma characteristics.

**Fig. 5:**
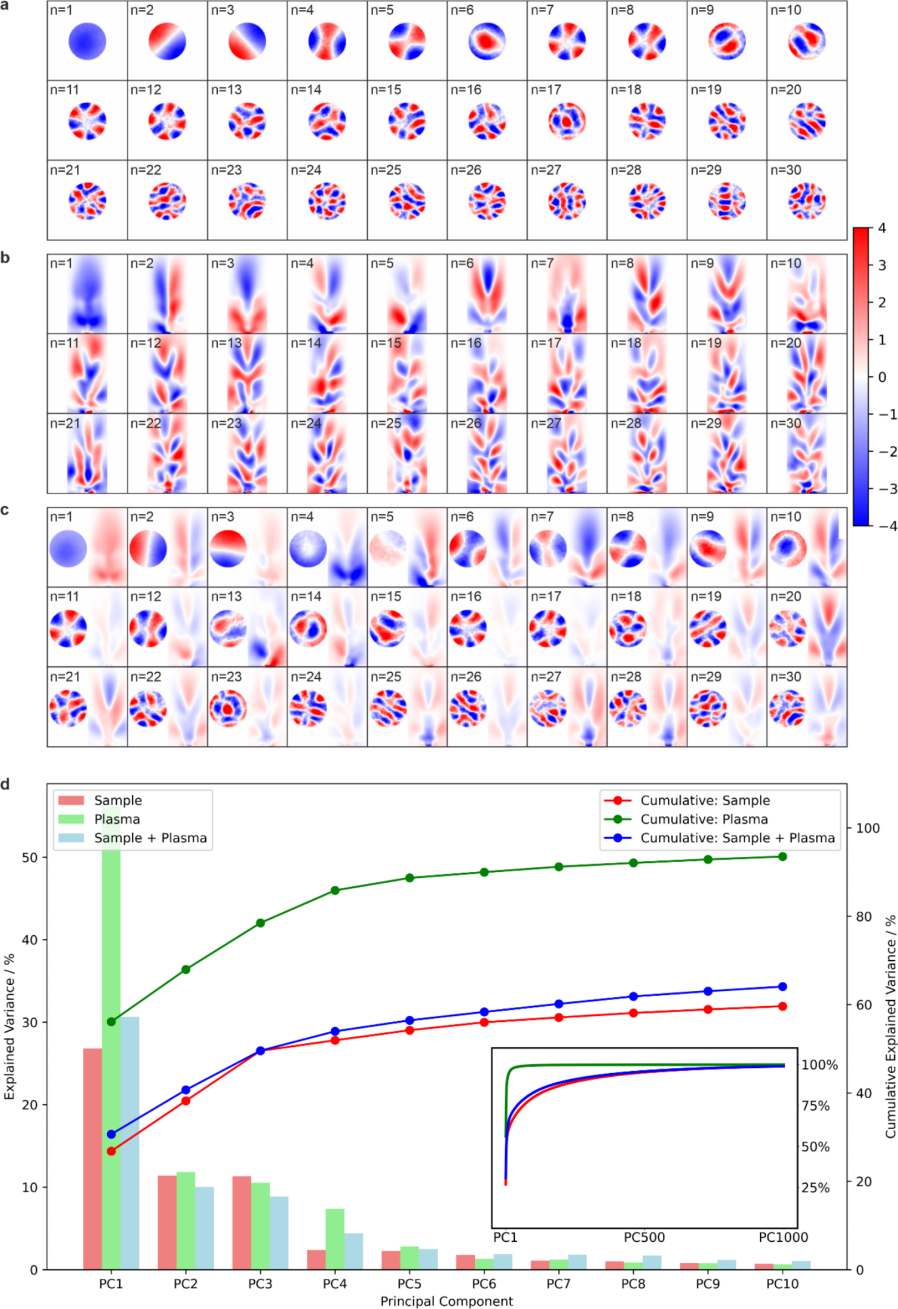
The first 30 principal components for **a**) sample only, **b**) plasma only, and **c**) sample and plasma combined, along with **d**) the explained variance (with the inset showing results to up the 1000th component).

Figure 5 shows the first 30 principal components for the cases of (a) sample only, (b) plasma only, and (c) sample and plasma combined. The results show that the complexity of the components increase as the component number increases, and components beyond the first ten become increasingly harder to interpret. The explained variance plot in (d) provides a visual representation of how much variance each principal component captures from the original data. The bars represent the individual explained variance for each component, and the cumulative line tracks the total explained variance as components are added, which can be used to identify how many components are needed to retain a desired proportion of the original information. The results indicate that the first four components can describe approximately 90% of the plasma variance and 50% of the sample variance, implying that the sample images include higher spatial complexity. An important observation is that the order of the components for the sample and the plasma changes when they are combined into a single image. For example, (a) *n* = 4 is similar to the sample in c) *n* = 6, and (b) *n* = 3 is similar to the plasma in (c) *n* = 7. In some cases, the orientation changes, for example a) *n* = 2 and *n* = 3 compared to the sample in c) *n* = 2 and *n* = 3. The reordering and rotation of components in the combined images is due to the correlations automatically being included in the PCA calculation. In addition, although all components are orthogonal, the plasma components in c) *n* = 20 to *n* = 30 are similar and symmetrical, whilst the sample components in c) *n* = 20 to *n* = 30 are distinct and asymmetrical. This indicates that sample features that are below a threshold size (approximately 5 μm in this case) result in only minimal changes to the appearance of the plasma.

**Fig. 6:**
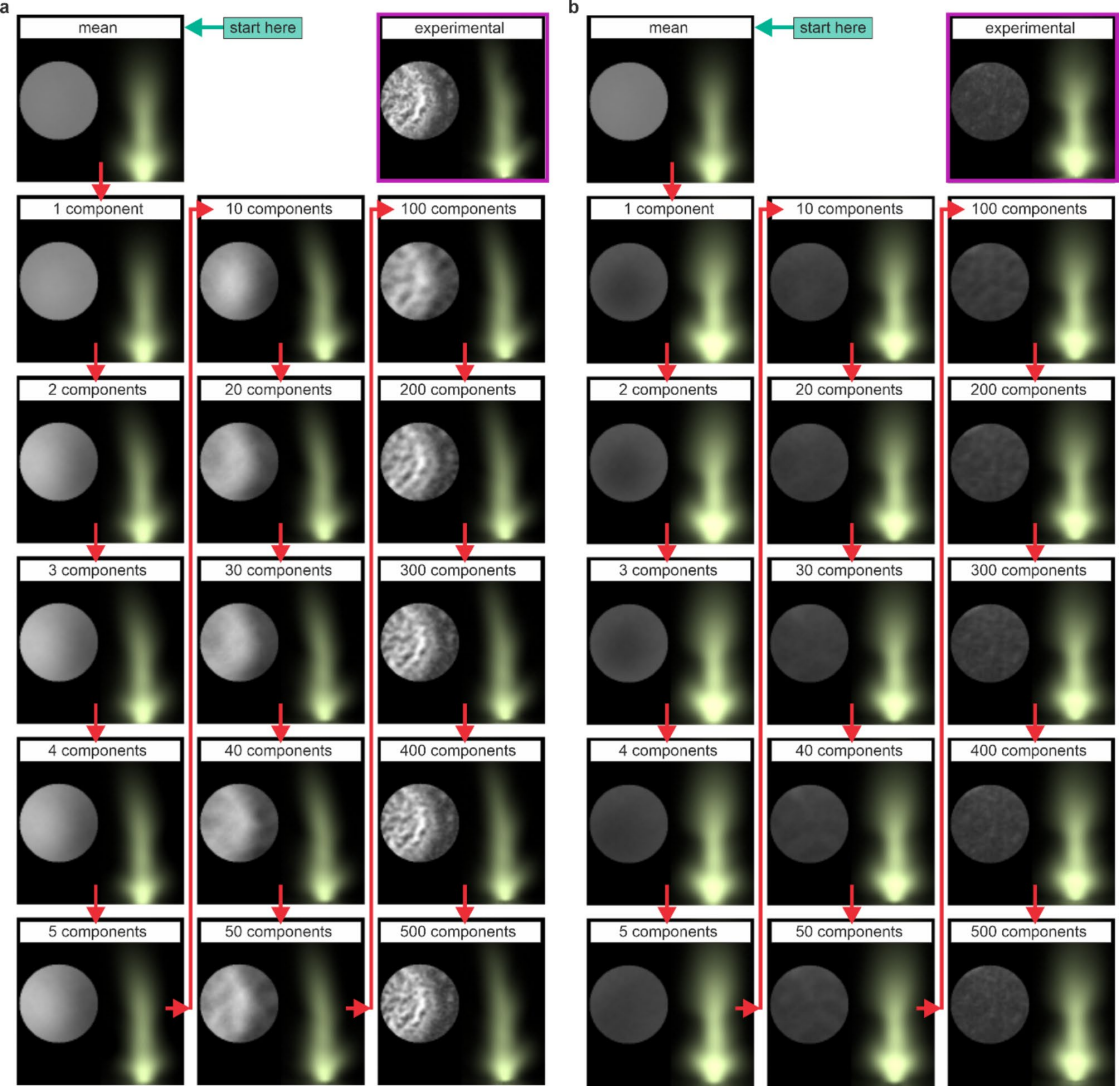
Constructed images when using increasing numbers of components, for a sample with **a**) stronger features on the left and **b**) with few surface features.

To show the effect of increasing the number of components used in a constructed image, Fig. 6 shows examples of images constructed for samples with (a) stronger features on the left and (b) with few surface features. Both cases show the progression from the mean image through to the use of 500 components and show the associated experimental image. Whilst both the constructed sample and plasma images closely match the experimental result when 500 components are used, the constructed plasma becomes more similar to the experimental result after fewer components, with the overall shape being formed after just a few components.

### Predicting the plasma appearance from the sample

**Fig. 7:**
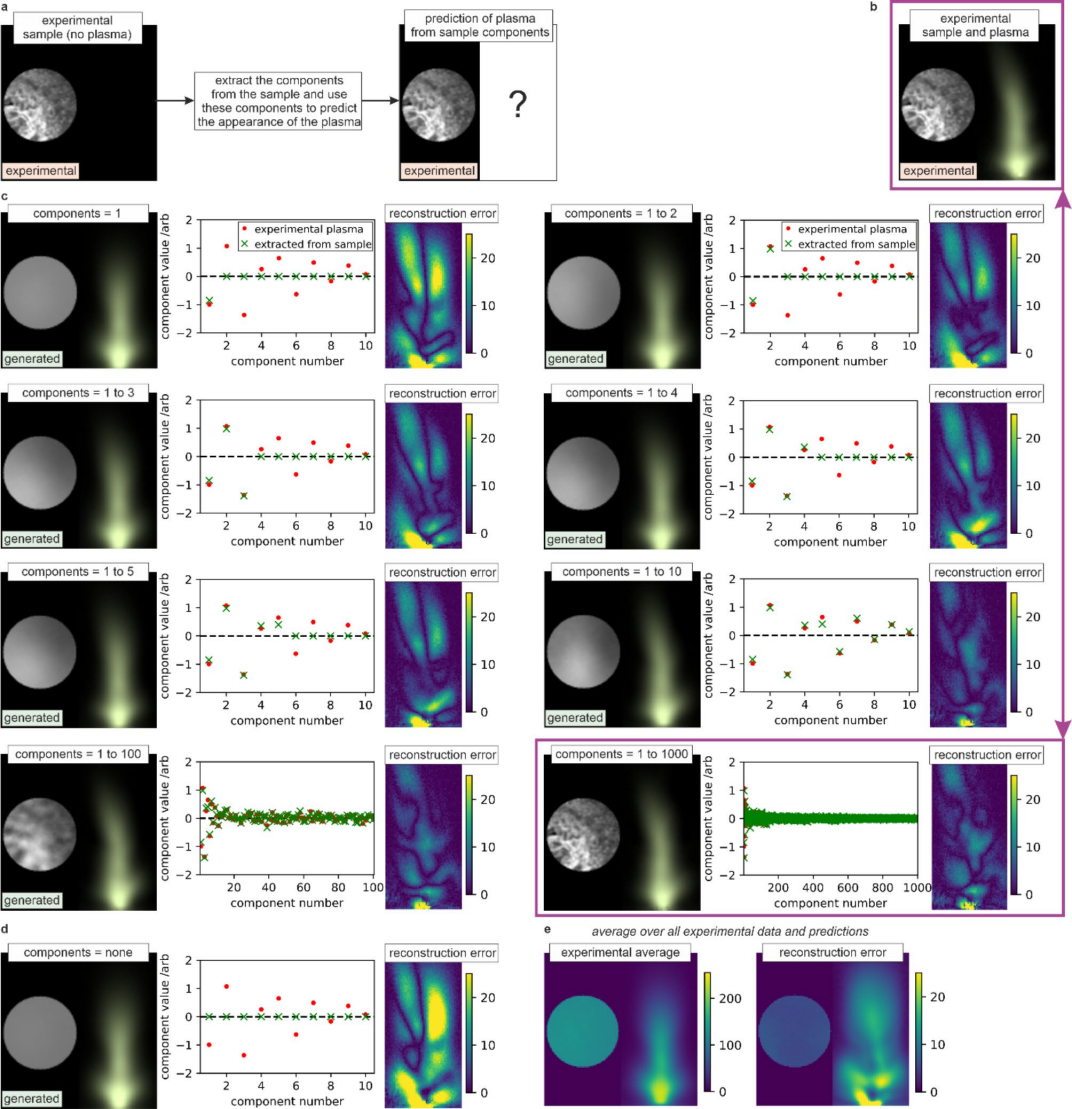
**a**) Concept of predicting the appearance of the plasma from the sample, with **b**) ground truth, **c**) predictions for different numbers of components, **d**) the mean, and **e**) average reconstruction error for all experimental data. Reconstruction errors are shown in terms of pixel value.

The principal components corresponding to the paired images (i.e., sample and plasma combined) indicate the correlations between features in the sample and the appearance of the plasma. It is therefore possible to predict the appearance of the plasma directly and exclusively from an image of the sample. Figure 7 demonstrates this capability, by predicting the plasma directly from a sample that was not used in the dataset for calculating the principal components. Figure 7a) shows a schematic of this concept, where the plasma is removed hence leaving only the sample visible, and the component values are extracted from the sample via PCA projection and these component values are then used to reconstruct (i.e., predict) the appearance of the plasma. Figure 7b) shows the experimental sample and plasma for reference (i.e., the ground truth). As the higher order modes contain higher granularity, it is interesting to compare the predictive capability when including different numbers of components. Figure 7c) shows the prediction for the plasma when combining 1, 2, 3, 4, 5, 10, 100 and 1000 predicted components, along with a plot comparing the component values for the experimental plasma image (which we are trying to predict) as red circles, and the components that were extracted from the sample (our predictions) as green crosses. Whilst it is possible to observe that the *n* = 2 mode bends the plasma to the left slightly and that the modes 6 to 10 make this bend more significant, it is the case that all additional modes only make subtle changes to the shape of the plasma. The purple box and lines correspond to the predicted plasma for 1000 components, and this compares strongly with the experimental result. In each case, the sample is reconstructed using the specified number of components and their predicted values, and hence it is also possible to see the granularity of the sample increasing as the number of modes are increased. In other words, around 10 components are needed to explain the plasma images but around 1000 components are needed to explain the sample images, which agrees with the cumulative explained variance results in Fig. 5d). Figure 7d) shows the result where all modes have a value of zero, and hence shows the mean. Figure 7e) shows both the average of all samples and the average prediction error, when this technique was applied to each experimental pair of sample and plasma (where the plasma to be predicted was not used in the PCA calculation in each case). The prediction errors are generally around 10% of the total plasma intensity, with the errors primarily around the base of the plasma. The observation that it is possible to predict the appearance of the sample directly and exclusively from the appearance of the sample provides strong evidence that the PCA approach shown here is effective at identifying the correlations between spatial features in the sample and spatial features in the plasma.

### Interpolation between experimental data

By representing each experimental image as a vector, where each vector contains the values of the principal components needed to reconstruct each experimental image, it is possible to interpolate between experimental data, effectively creating new, synthetic images. This interpolation technique allows for the exploration of the relationships between sample features and plasma shape in a continuous manner, potentially allowing further investigation into the dynamic interplay between these two imaging modalities.

**Fig. 8:**
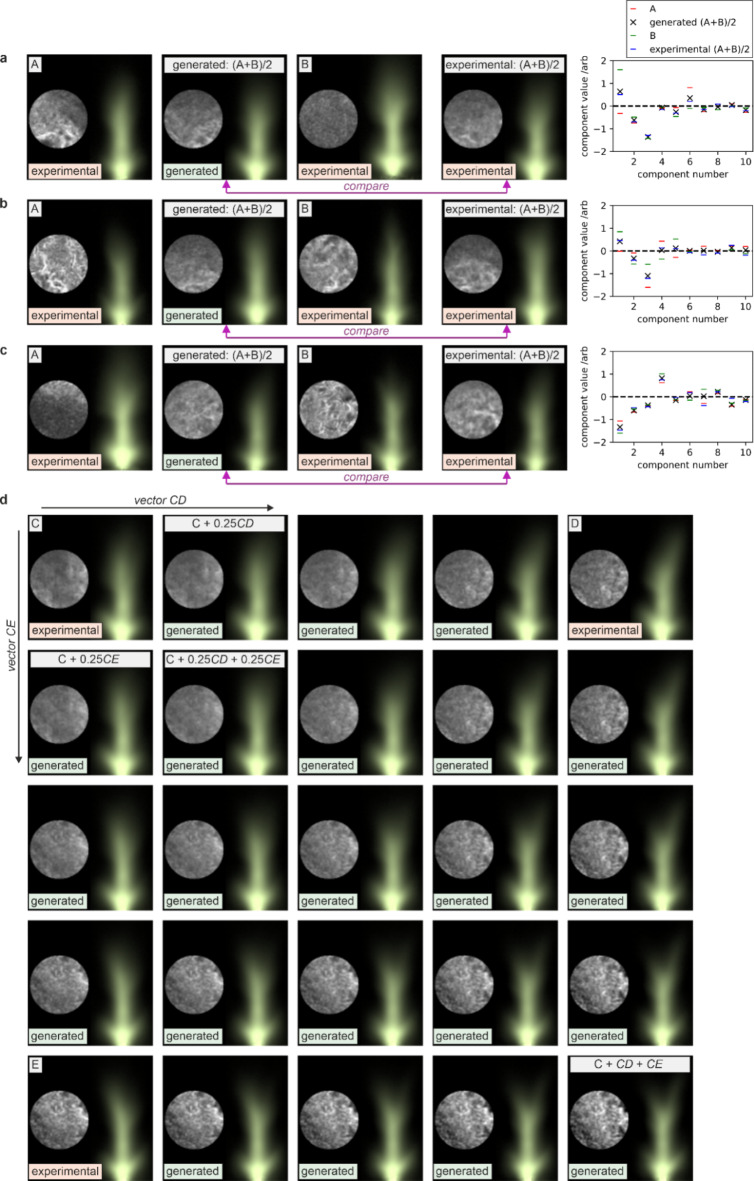
**a**-**c**) Images generated by interpolating mid-way between two experimental images as compared to the associated experimental images and **d**) generated images created through interpolation between three experimental images.

Figure 8a-c) shows a validation process for this technique, through presenting two experimental images (A and B) and generating an image that corresponds to the interpolated mid-point of the two images. Three examples are provided and, in each case, an experimental image corresponding to the mid-point is shown for comparison. While surface-to-plasma predictions using PCA are moderately effective, the plasma-to-surface predictions are less effective, which we attribute to a poorer correlation of higher-order surface features to the plasma image features. To demonstrate this capability further, Fig. 8d) shows three examples of experimental data (C, D, and E) along with associated interpolations, through the addition of ratios of the vectors CD (components C subtracted from components D) and CE (components C subtracted from components E). The interpolated images show a prediction for how the transformation of a sample would result in changes to the appearance of the generated plasma, hence offering an equivalent of partial differentials across the experimental data. The figure shows that the combination of a vector from a symmetrical plasma to a right-leaning plasma (CD) and a vector from a symmetrical plasma to a left-leaning plasma (CE) can produce a split plasma (C + CD + CE). This interpolation approach could therefore be used to further understand the association between specific changes in the sample and the resultant change to the plasma, and could be particularly valuable in scenarios where direct experimentation might be time-consuming or costly.

### Resemblance to Zernike polynomials

**Fig. 9:**
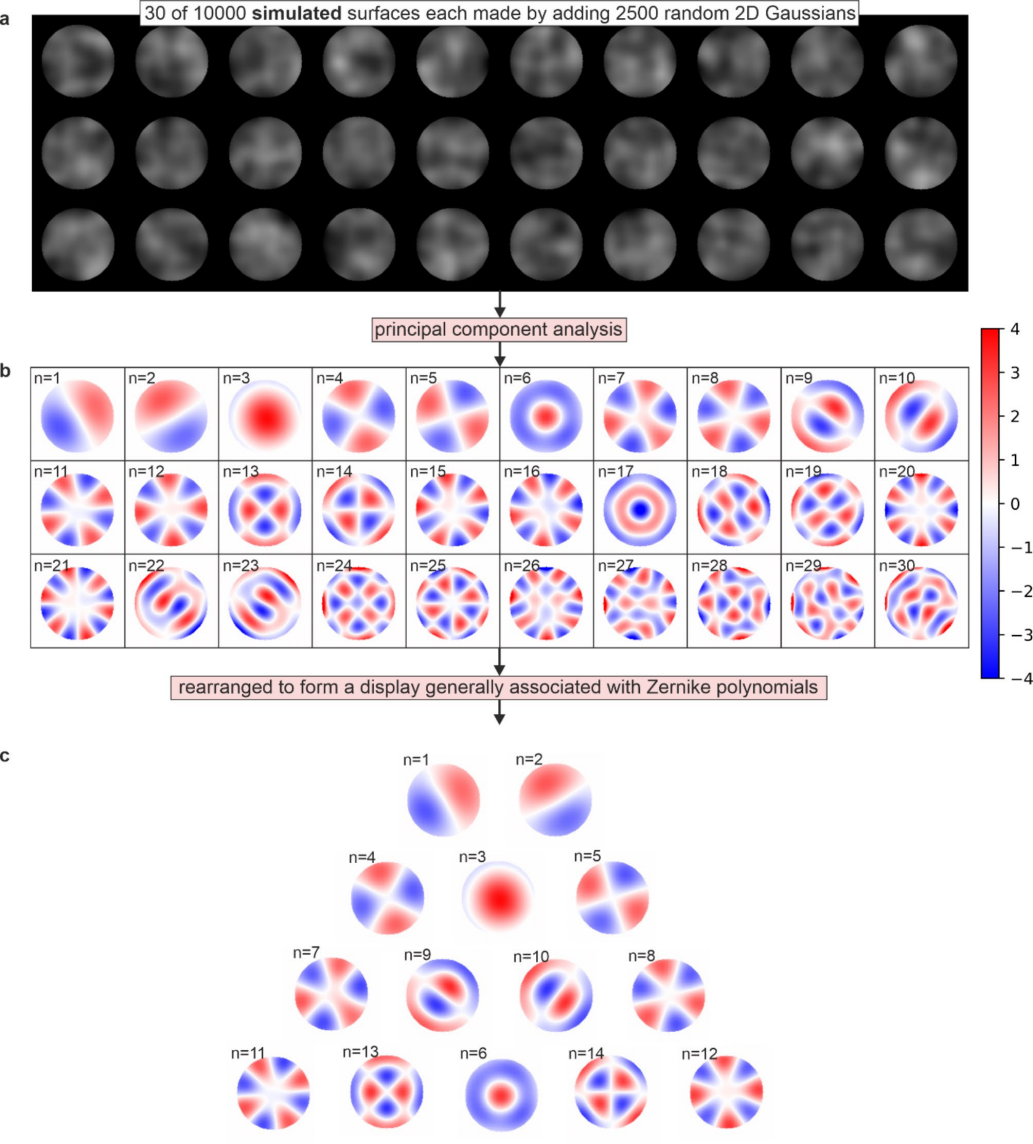
Explanation of the resemblance of surface morphology components to Zernike polynomials. Showing **a**) 30 examples of simulated surfaces, **b**) associated principal components, and **c**) a rearrangement of these components to form a display generally associated with Zernike polynomials.

It is interest to note that the surface morphology principal components in Fig. 5 resemble Zernike polynomial distributions. This phenomenon arises because Zernike polynomials form a complete orthogonal basis for functions defined over the unit disk. The 2D arrays corresponding to the experimental microscope images of the sample can be considered mathematically similar to a superposition of smooth, radially symmetric patterns that vary in spatial frequency and orientation, and therefore PCA decomposes these images into orthogonal modes that resemble Zernike polynomials. To demonstrate this more clearly, Fig. 9 shows the effect of simulating the appearance of the sample surface 10,000 times (each made by adding together 2500 2D Gaussian distributions with random width and position, converting this into a black and white image, and cropping into a circular shape) and then performing PCA on this simulated dataset. As the process for creating these simulated samples is random, the principal components very closely resemble Zernike polynomials. Figure 9 shows (a) 30 examples of simulated samples, (b) the associated first 30 principal components, and (c) a rearrangement of these components to form a display generally associated with Zernike polynomials. This analysis underscores how the inherent mathematical properties of PCA align with the structural characteristics of Zernike polynomials in representing smooth, radially symmetric image features.

## Discussion

PCA is most effective when the underlying relationships between variables are linear, as the transformation it performs captures linear variance. Therefore, if the data involves complex nonlinear patterns, such as the case here with a nonlinear light-matter interaction, the application of PCA may lead to information loss. Whilst nonlinear dimensionality reduction techniques, such as t-SNE (t-Distributed Stochastic Neighbour Embedding), could be applied, this might have the disadvantages of requiring additional computation and having lengthy parameter optimisation, whilst also reducing explainability. In this proof-of-concept demonstration of dimensionality reduction for identifying correlations between plasma features and sample features, PCA was chosen due its simplicity and speed in application, its explainability, and that it is well-known mathematical technique. However, despite these limitations, the interpolation validation in Fig. 8a) shows that the PCA approach is shown to be an effective approach in this case.

In this case, the highest laser pulse energy available was used, to ensure that the plasma size was maximised and hence minimise the constraints on imaging. Investigation of the size and shape of plasma formation under different pulse energies is suggested as a focus for further work. In this work, the appearance of the sample was correlated with the appearance of the plasma. However, future work could focus on the correlation between the 3D profile of the sample surface with the plasma, for example by using a surface diagnostic interferometric imaging technique to measure the sample profile between laser pulses. Whilst this work has identified correlations between the appearance of the sample and the appearance of the plasma, it is important to consider that nonlinear effects that do not correspond directly to the sample surface, such as nonlinear focussing and ionisation of the air, may also contribute to the appearance of the plasma. However, in this work, these effects are expected to be constant for all experimental data, and hence their effects are likely encoded into the identified correlations.

In this experiment, due to the sub-micron average depth of machining and limited spatial overlapping of machined regions, it is assumed that there were no cases where laser light entered craters deep enough to affect the plasma imaging. Silicon laser machining can result in considerable nanoparticles and debris, as observed in some images, and hence the PCA approach may have identified the correlations for both the appearance of the sample and the adjacent sample debris, against the appearance of the plasma. Future work could focus on untangling the different effects on the plasma from both 3D surface modulations and laser-machined debris. Whilst the proposed PCA approach can enable a level of explainability that might be consider beyond what is offered by deep learning approaches, the interpretation of higher-order modes remains a challenge. We propose two approaches for further investigation. Firstly, to gain clearer insights into the intricate spatial patterns, we propose decomposing higher-order modes into their constituent spatial frequencies or applying feature importance analysis to identify the most significant regions influencing these modes. Additionally, correlating these higher-order modes with physical parameters of the laser machining process, such as laser fluence or pulse duration, could reveal hidden relationships between these parameters and the observed spatial variations. Second, to decouple systematic artifacts from real physical effects, we recommend systematically varying experimental parameters such as laser alignment and objective positioning to examine their influence on the PCA results.

We have demonstrated the application for single laser pulses, and hence our results do not account for the inter-pulse effects that become significant at the high repetition rates used in industrial laser ablation systems. For example, heat accumulation could alter the material properties of the machined sample, potentially affecting plasma generation and its spatial characteristics. Likewise, plasma shielding, where the generated plasma absorbs or deflects subsequent laser pulses, may also interfere with the imaging of the plasma. Whilst there are methods for potentially minimising these effects, such as via sample cooling or using time-resolved imaging, we anticipate that the shape of the plasma for high repetition rate lasers may include information associated with inter-pulse effects, such as the magnitude of heat accumulation and plasma shielding, and hence this approach could possibly be directly applied onto a high repetition rate laser ablation system. However, further investigation is clearly needed.

Previous work has shown that neural networks can be used to predict the appearance of the sample directly from the appearance of the plasma^17^. This is surprising given the direction of imaging, as the plasma is observed from a direction that is perpendicular to the normal to the sample surface. Despite this, only a single image is needed to transform from sample to plasma, or vice-versa. This could be considered similar to achieving 3D tomographic imaging from a single projection. However, unlike neural networks, which are extremely challenging to interpret, PCA can be used to provide an intuitive mathematical explanation of why this transformation is possible, and this explanation is an important outcome of the work presented here. Analysis of the *n* = 2 and *n* = 3 modes (see Fig. 4c)) provides a key component of this explanation. The *n* = 2 mode shows that the presence of machined features on the left of the sample causes the plasma to be brighter on the left. The *n* = 3 mode shows that the position of features in the top-bottom direction results in slight changes to the height of the plasma and width of the neck. As the plasma image recorded on the camera is the integral of the plasma emission along the direction normal to the camera, it is therefore surprising that there is a mode equivalent to the *n* = 3 mode. It is hypothesised that the *n* = 3 mode exists purely due to asymmetries in the laser machining setup, with the most likely reason being an asymmetry in the laser spatial intensity profile, although other asymmetries in the alignment of the camera or the imaging objective could also be a cause. Nevertheless, if this hypothesis is true, then the ability to predict the appearance of the plasma from the sample (and vice-versa) is only possible if asymmetries are present in the experimental setup. As the plasma generation process is highly nonlinear, it is possible that even very small asymmetries are sufficient to allow for this imaging technique to exist, and perhaps it is even practically impossible to align the experimental system to prevent this technique from working. An important conclusion is therefore that the plasma emission, even when recorded from a single perpendicular imaging plane, contains a significant amount of information about the features and surface profile of the target sample. In general, laser machining produces a strong plasma emission, and hence the sample cannot often be observed directly during the machining process. Monitoring the plasma, instead of the sample itself, therefore offers the prospect to be an extremely useful monitoring technique for industrial and academic laser materials processing, and PCA could be used to process these images and provide new insights into the physical mechanisms that occur during femtosecond laser-matter interactions.

## Conclusions

Principal component analysis has been applied to identify the association between features on the surface of the sample and the spatial profile of the generated plasma, when femtosecond laser pulses are used to machine silicon. The analysis shows that the shape of the plasma depends strongly on the position of features within the laser focal region on the sample, and that the correlations between feature position and plasma profile can be identified, hence unlocking the potential for data-driven scientific discovery in the field of femtosecond light-matter interactions. Given that the sample cannot generally be observed directly during laser machining due to the intense plasma that is produced, the presented method also demonstrates a key innovation for the optimisation of laser materials processing.

## Data Availability

The datasets used and/or analysed during the current study are available from the corresponding author on reasonable request.
